# Diurnal variations of fever in children: results from an app-based observational study

**DOI:** 10.1186/s12887-026-07432-y

**Published:** 2026-08-05

**Authors:** Yvonne Beerenbrock, Ricarda Möhler, Larisa Rathjens, Ekkehart Jenetzky, David D. Martin

**Affiliations:** 1 Faculty of Health (School of Medicine), Chair of Medical Theory, Integrative and Anthroposophic Medicine, Institute for Integrative Medicine, Witten/Herdecke University, Gerhard-Kienle-Weg 4, Herdecke, 58313 Germany; 2 Department of Child and Adolescent Psychiatry and Psychotherapy, University Medical Center of the Johannes-Gutenberg-University, Mainz, Germany; 3 Tübingen University Children’s Hospital, Eberhard-Karls University, Tübingen, Germany

**Keywords:** Fever, Circadian rhythm, Childhood fever, Mobile health, Participatory research

## Abstract

**Background:**

Fever is a frequent concern in early childhood. However, its management remains inconsistent, driven in part by persistent “fever phobia” and heterogeneous advice. Although body temperature follows a well-established circadian rhythm, the diurnal expression of fever in young children is insufficiently described. We aimed to characterise 24-hour patterns of febrile temperatures in children aged 0–7 years using real-world data from a participatory mobile health registry (FeverApp), with specific attention to age and antipyretic use.

**Methods:**

We analysed 28,620 fever episodes recorded in the FeverApp registry between September 2019 and September 2023, comprising 12,454 children (median age 14 months) and 122,890 time-stamped temperature measurements. Mean hourly temperatures were calculated by pooling all eligible entries within hourly bins and were stratified descriptively by age group and recorded antipyretic use, without consideration of dependency. Between-group differences were summarised descriptively using mean differences.

**Results:**

Mean temperature demonstrated a clear diurnal pattern, reaching a nadir at 06:00 (38.25 °C) and a late-evening peak at 23:00 (38.93 °C), corresponding to an observed trough-to-peak difference of 0.68 °C. Infants (< 12 months) showed lower entry-level mean temperatures than older children (1–7 years), with differences smallest around the morning trough and more pronounced later in the day. Recordings close to documented antipyretic administration had higher mean temperatures throughout the day, consistent with confounding by indication, while the overall diurnal pattern remained preserved. High-fever measurements (≥ 39 °C) were least frequent in the early morning (05:00–08:59) and most frequent during late evening/night (21:00–00:59).

**Conclusions:**

Fever in young children should be interpreted as a dynamic 24-hour pattern rather than as a single static temperature value. In this app-based observational cohort, recorded febrile temperatures followed a clear aggregate time-of-day pattern, with lower values in the early morning and higher values later in the day. This temporal context may help clinicians and caregivers interpret fever more realistically: a low morning value should not be used as reassurance in isolation, while late-day increases may partly reflect expected circadian dynamics. Because the analysis is descriptive and entry-level, these findings should not be interpreted as individual fever trajectories, child-level risks, or inferential estimates.

**Trial registration:**

The FeverApp registry is registered in the German Clinical Trials Register (DRKS), number DRKS00016591, registered 07 February 2019 (https://drks.de/search/en/trial/DRKS00016591), as a non-interventional observational study protocol.

**Supplementary Information:**

The online version contains supplementary material available at 10.1186/s12887-026-07432-y.

## Background

Fever remains among the most common reasons for consultation in pediatric emergency departments [1] and illustrates how caregiver behaviour is shaped by cultural beliefs and cognitive biases, most notably the persistent phenomenon of “fever phobia” [2]. Despite robust evidence that most childhood fevers are self-limited and benign, anxiety about elevated body temperature continues to drive premature or excessive interventions. Practices such as alternating antipyretics or administering medication at the first sign of fever or with the sole aim of lowering the temperature remain common, although supportive evidence is limited [3]. This discrepancy underscores the ongoing need for accessible, evidence-based guidance that situates fever within physiological norms and supports appropriate decision-making [4].

Chronobiology provides an important framework for such contextualisation. In healthy individuals, core body temperature follows a well-described circadian rhythm, typically fluctuating by around 1 °C over 24 h [5, 6]. The nadir usually occurs in the early morning (approximately 04:00–06:00), followed by a rise toward an afternoon or early-evening peak [5, 6]. This rhythm is closely linked to hormonal cycles (e.g., cortisol and melatonin) and central thermoregulatory pathways [7]. In U.S. national triage data, fever detection was substantially lower in the morning than later in the day, and high-grade fever (> 39 °C) was comparatively uncommon before 09:00 [8]. Although atypical courses exist (e.g., persistent fever or irregular trajectories), diurnal variation remains a common feature in uncomplicated viral and bacterial illness [9].

Despite these established principles, diurnal patterns of pediatric fever remain insufficiently characterised outside institutional settings. High-frequency, time-stamped temperature data from the home environment have traditionally been scarce; app-based registries now enable ecological capture of fever trajectories at scale [10, 11]. Applications such as FeverApp allow caregivers to document temperature, symptoms, and medication use in real time, generating ecologically valid datasets for chronobiological and clinical analyses [11]. Beyond functioning as a digital fever diary, FeverApp was designed as a participatory research platform, enabling caregivers to contribute structured real-world data while receiving guideline-based support for fever management [12]. This approach aligns with broader developments in public health and digital citizen science, including efforts to strengthen public trust and engagement in real-world evidence generation [13].

From a medical informatics perspective, FeverApp operationalises a scalable ecological momentary assessment (EMA) registry model. Caregivers record structured, time-stamped entries on symptoms, temperature, and medication use via smartphone, with synchronisation to an academically governed database for secondary analyses. In contrast to many standalone fever diary applications, the key innovation lies in combining guideline-based decision support with the generation of pseudonymised real-world data that can inform pediatric and digital health research.

In the present study, we analyse FeverApp registry data to describe temporal dynamics of fever in children aged 0–7 years. Our primary objective is to characterise the aggregate 24-hour temperature pattern during febrile episodes, with specific attention to age strata and recorded antipyretic use. Rather than estimating individual fever trajectories, we summarise how caregiver-recorded temperature entries are distributed across the day in a large real-world registry. We also explore whether the expected lower morning and higher late-day temperature pattern remains visible during febrile illness and whether this aggregate pattern differs by age group or recorded antipyretic exposure.

A more precise understanding of these patterns is directly relevant for clinical practice. It supports a more nuanced interpretation of fever measurements, particularly early-morning readings, and can help families form realistic expectations regarding daily fluctuations. Moreover, this study illustrates how citizen-engaged, app-based data collection can generate clinically meaningful insights into pediatric health phenomena that are difficult to capture through conventional clinical workflows.

## Methods

### Study design and data source

This study is a retrospective observational analysis based on data from the FeverApp registry, an ongoing app-based cohort designed to capture pediatric febrile episodes in real-world home settings. FeverApp is a freely available smartphone application developed by pediatric specialists and researchers and launched in Germany in 2019. The app serves two purposes: (i) providing caregivers with evidence-based information and guidance on fever management, and (ii) enabling structured, standardised documentation of febrile episodes for research. During the study period, FeverApp was available to families throughout Germany; however, recruitment and use were not population-based. Families accessed the app through participating pediatric practices and study-related dissemination channels, and the registry therefore represents a Germany-based app-using cohort rather than a representative sample of all children in Germany.

During a febrile episode, parents or legal guardians can record the child’s body temperature, associated symptoms, administered antipyretics and other medications, and relevant contextual information. All entries are time-stamped and synchronised in pseudonymised form to a central research database (the FeverApp registry), allowing analyses of fever trajectories under naturalistic conditions. For the present analysis, the temperature-entry dataset (“loops”) was merged with the medication dataset. An antipyretic-use indicator was derived from medication entries and linked to the corresponding temperature data to enable stratified analyses by recorded antipyretic administration.

Participation is voluntary and requires informed digital consent obtained during the in-app registration process. The study protocol, including procedures for data protection, pseudonymisation, and ethical oversight, was approved by the responsible institutional research ethics board (see Declarations). The registry is registered in the German Clinical Trials Register (DRKS) as a non-interventional observational cohort (DRKS0001659, registered 07 February 2019, https://drks.de/search/en/trial/DRKS00016591). Data processing was conducted in accordance with applicable data-protection regulations, including the General Data Protection Regulation (GDPR).

### Participants and inclusion criteria

We analysed febrile events recorded in children aged 0–7 years in the FeverApp registry. For clarity, we distinguish between a fever episode (the overall febrile event) and the clinical stages of fever that may occur within an episode. Operationally, a fever episode was defined as a sequence of fever-related entries without a gap exceeding 48 h; a pause of > 48 h between entries was treated as the boundary to a new episode.

Within a single fever episode, children may pass through classic phases (rise, plateau/peak, and defervescence). We did not classify recordings into these phases of a single episode; instead, we analysed the time-stamped temperature trajectory independent from any stage. We included all fever episodes recorded between September 2019 (registry launch) and September 2023 that contained at least one temperature measurement ≥ 38.0 °C (100.4 °F). Episodes temporally associated with routine vaccinations were retained.

To improve measurement comparability and to reflect age-specific recommendations applied in this study, we restricted the dataset to rectal temperature measurements in infants (< 12 months) and tympanic measurements in children aged 1–7 years. Measurements obtained from other sites (e.g., axillary or oral) were excluded. This approach is consistent with German guidance, which commonly recommends rectal measurement in infants and tympanic measurement in older children, while oral measurement is generally reserved for adolescents. As this study is based on a registry with passive ecological momentary assessment, active follow-up of individual participants was not conducted and is not applicable to this study design.

### Data collection and variables

For each episode, FeverApp enabled structured, time-stamped documentation of body temperature (recorded to one decimal place), the time of measurement (local time), and caregiver-reported antipyretic administration. Beyond the numeric temperature value, the app allows caregivers to record accompanying symptoms, general condition and warning signs, illness duration, healthcare contacts, and medication use. These contextual data were not used to assign diagnoses in the present analysis.

The primary variables for this analysis were: (1) time of day of the temperature recording, (2) age group (infants (< 12 months) and older children (1–7 years)) and (3) antipyretic use. Antipyretic use was operationalised based on medication entries recorded within ± 1 h of the corresponding temperature measurement. Recorded antipyretics predominantly comprised paracetamol and ibuprofen. The exact dose, formulation, route of administration, adherence, and pharmacodynamic timing beyond the ± 1-hour exposure window were not analysed.

FeverApp also allows documentation of broader clinical context (e.g., symptoms, healthcare utilisation, and illness duration). These elements have been reported elsewhere [14] and were not the focus of the present analysis. Physician-confirmed diagnoses, laboratory or microbiological results, and sepsis criteria were not available in the analysed dataset. Consequently, episodes could not be differentiated into upper respiratory tract infections, urinary tract infections, gastroenteritis, sepsis, or other aetiological categories. Pre-existing neurological conditions, hypothalamic dysfunction, structural brain abnormalities, or other disorders potentially affecting thermoregulation were likewise not systematically captured. Such children could therefore not be analysed separately or excluded with certainty.

### Outcome measures and statistical analysis

The primary outcome was the diurnal (time-of-day) pattern of body temperature during febrile episodes. To quantify and visualise this pattern, each temperature recording was assigned to an hourly interval (00:00–00:59, 01:00–01:59, …, 23:00–23:59), and the entry-level mean temperature was calculated for each hour across all eligible recordings.

All analyses in the revised manuscript are descriptive and entry-level. No weighting by child, fever episode, or family was applied. Therefore, the displayed fever curves represent the average temperature at each time point across all eligible entries from all children, not the trajectory of any individual child. Because some children contributed only one entry whereas others contributed multiple entries at the same time or across different days, the measurements are not statistically independent.

To visualise the distribution underlying the hourly mean curves, we additionally generated a binned density heatmap of the entry-level temperature recordings (Supplementary Figure S1). Temperature values, recorded to one decimal place, were cross-tabulated by hour of day and 0.1 °C temperature bins and displayed as relative frequencies. This heatmap was used as a descriptive visualisation only. It did not incorporate weighting or clustering by child, fever episode, or family and was therefore not used for inferential interpretation.

For prespecified secondary descriptions, separate 24-hour curves were generated for age group (infants < 12 months vs. children aged 1–7 years) and recorded antipyretic exposure (measurements with vs. without a documented antipyretic within ± 1 h of the temperature entry). Group differences were summarised as observed entry-level mean differences. These comparisons are not interpreted as independent-sample tests.

In addition to mean trajectories, we evaluated the time-of-day distribution of clinically relevant fever thresholds. Temperatures ≥ 39.0 °C were classified as high fever and ≥ 40.0 °C as very high fever. Frequencies and observed proportions were summarised across six prespecified 4-hour intervals (01:00–04:59, 05:00–08:59, 09:00–12:59, 13:00–16:59, 17:00–20:59, 21:00–00:59). Analyses were conducted using IBM SPSS Statistics (version 28, IBM Corp., Armonk, NY, USA). Missing data were not imputed.

### Bias and error considerations

As this observational analysis was based on caregiver-entered data, several potential sources of bias and measurement error were considered. The restriction to rectal measurements in infants and tympanic measurements in older children improves comparability within age strata, but it also creates complete confounding of measurement site with age group. Differences in thermometer type, device calibration, and caregiver technique could not be standardised.

The timing of temperature recordings was caregiver-driven and may therefore be affected by sampling bias. Measurements are more likely to be taken when caregivers anticipate rising fever, when the child is awake, or when symptoms cause concern. Recording density was lower during night-time and early-morning hours, which may influence mean estimates at specific time points.

Antipyretic administration was self-reported and may be incompletely captured. The absence of a medication entry may indicate true non-use or missing documentation. Antipyretic exposure was defined by medication records within ± 1 h of the corresponding temperature measurement; dose, formulation, route, adherence, and non-pharmacological interventions such as cooling wraps were not analysed.

## Results

### Participant characteristics and dataset overview

A total of 122,890 temperature recordings from 28,620 distinct fever episodes in 12,454 children were included. This corresponds to a mean of approximately 2.3 episodes per child, 4.3 temperature recordings per episode, and 9.9 temperature recordings per child. These observation levels are reported to make the hierarchical and heterogeneous data structure transparent; however, the revised analyses do not treat repeated entries as independent child-level observations. Participant characteristics are summarised in Table 1. The cohort consisted predominantly of young children, with a median age of 14 months (IQR 0–30), a mean age of 21 months (SD 18), and an age range of 0–84 months. Overall, 6,569 children were male (52.7%) and 5,885 were female (47.3%).

**Table 1:** Sample characteristics of the FeverApp dataset (September 2019–September 2023)

Characteristics	Value
Study Period	September 2019 – September 2023
Total number of children	12,454
Age range	0 to 7 years
Median age (IQR)	14 months (IQR 0–30 months); mean 21 months (SD 18); range 0–84 months
Sex distribution	5,885 female (47.3%) / 6,569 male (52.7%)
Total number of fever episodes	28,620
Total number of temperature readings	122,890
Accepted measurement methods	Rectal (< 12 months) and tympanic (1–7 years)
Episodes with antipyretic use	3,949 (14.6% of 27,108 episodes with medication data; 1,512 missing)
Episodes without antipyretic use	23,159 (85.4% of 27,108 episodes with medication data; 1,512 missing)
Median peak temperature per episode	39.2 °C (IQR: 38.6–39.7 °C)
Children < 12 months	5,531 children (44.4%)

Baseline characteristics of the FeverApp cohort and registry dataset included in the present analysis. Values are reported as n (%), median (IQR), or mean (SD), as appropriate

*Abbreviations*: *IQR* interquartile range, *SD* standard deviation

Medication information, including no medication, was available for 27,108 episodes. Among these, 3,949 episodes (14.6%) included a recorded antipyretic entry, whereas 23,159 episodes (85.4%) had no antipyretic documented; medication data were missing for 1,512 episodes. The median peak temperature per episode was 39.2 °C (IQR 38.6–39.7 °C).

Before summarising hourly mean temperatures, the entry-level distribution of recorded temperatures was inspected using a binned density heatmap (Supplementary Figure S1). This descriptive visualisation showed that most app-recorded measurements clustered between approximately 38 °C and 40 °C across the 24-hour period, with a visible shift toward higher temperature bins in the late evening and night. The density of recordings at or above 39 °C appeared lowest during the early morning and increased later in the day, consistent with the descriptive threshold analysis. Because each entry contributed equally, the heatmap illustrates the registry-level distribution of recorded measurements and should not be interpreted as an individual fever trajectory or as a child-level risk estimate.

### Diurnal pattern of fever in the overall sample

**Fig. 1:**
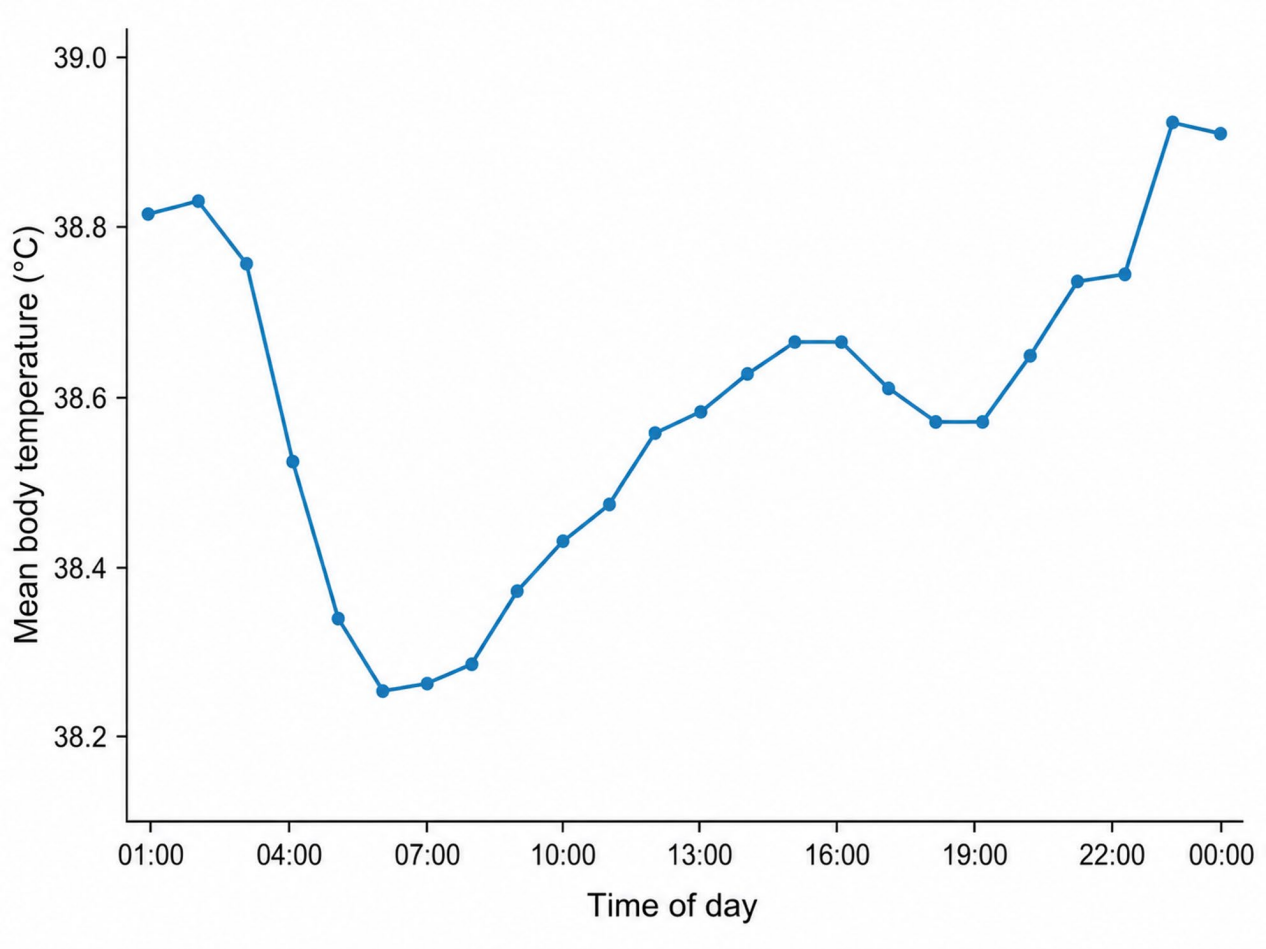
Diurnal fever curve in the overall sample. Entry-level mean body temperature by hour of day (local time) across all eligible temperature recordings. The line represents aggregate registry-level means; it should not be interpreted as an individual fever trajectory or as a cluster-corrected estimate

Febrile temperatures showed a distinct diurnal pattern across the recorded entries. As illustrated in Fig. 1, entry-level mean body temperature varied systematically over the 24-hour cycle, with the lowest values occurring in the early morning and a gradual increase toward a late-evening maximum. Mean temperature reached its nadir at 06:00 (38.25 °C) and peaked at 23:00 (38.93 °C), corresponding to an observed trough-to-peak difference of 0.68 °C. Detailed hourly descriptive statistics are provided in Table 2.

**Table 2:** Hourly summary statistics of paediatric body temperature during febrile episodes (overall sample)

Time	*N* (entries/hour)	Mean temp (°C)	Upper 95% CI	Upper 95% CI – Mean	Lower 95% CI	Mean – Lower 95% CI
1:00 AM	1,302	38.82	38.87	0.05	38.78	0.04
2:00 AM	1,159	38.83	38.88	0.05	38.78	0.05
3:00 AM	1,143	38.76	38.81	0.05	38.71	0.05
4:00 AM	2,283	38.52	38.56	0.04	38.48	0.04
5:00 AM	4,953	38.33	38.36	0.03	38.31	0.02
6:00 AM	6,778	38.25	38.27	0.02	38.23	0.02
7:00 AM	7,070	38.26	38.28	0.02	38.24	0.02
8:00 AM	6,304	38.28	38.31	0.03	38.26	0.02
9:00 AM	5,619	38.36	38.39	0.03	38.34	0.02
10:00 AM	5,811	38.42	38.44	0.02	38.40	0.02
11:00 AM	6,014	38.46	38.49	0.03	38.44	0.02
12:00 PM	6,171	38.55	38.57	0.02	38.53	0.02
1:00 PM	6,505	38.58	38.60	0.02	38.56	0.02
2:00 PM	6,584	38.63	38.66	0.03	38.61	0.02
3:00 PM	6,472	38.67	38.69	0.02	38.65	0.02
4:00 PM	7,228	38.67	38.69	0.02	38.65	0.02
5:00 PM	9,181	38.61	38.63	0.02	38.59	0.02
6:00 PM	9,404	38.57	38.58	0.01	38.55	0.02
7:00 PM	7,491	38.57	38.59	0.02	38.55	0.02
8:00 PM	5,744	38.64	38.66	0.02	38.61	0.03
9:00 PM	4,004	38.73	38.76	0.03	38.69	0.04
10:00 PM	2,431	38.74	38.78	0.04	38.70	0.04
11:00 PM	1,732	38.93	38.97	0.04	38.88	0.05
12:00 AM	1,492	38.92	38.97	0.05	38.88	0.04

Mean body temperature (°C) aggregated by hour of day across all eligible temperature recordings. For each hourly bin, the table reports the mean, upper and lower 95% confidence interval (CI), and the CI half-widths (upper 95% CI – mean; mean – lower 95% CI)

*Abbreviations*: *CI* confidence interval, *N* number of temperature entries per hour

When compared with established physiological baselines, the overall shape of the pediatric fever curve resembles the canonical circadian rhythm of core body temperature in healthy individuals, albeit shifted upward due to the febrile state [5, 6]. The trough-to-peak amplitude in our dataset was 0.68 °C, broadly consistent with the circadian range reported in normothermic populations.

However, the timing of the maximum differed from the canonical pattern. In healthy individuals, the acrophase typically occurs in the late afternoon or early evening (around 17:00–18:00), followed by a gradual decline toward the nocturnal nadir [5, 6]. In contrast, febrile children in our cohort continued to show rising mean temperatures beyond this expected turning point, with peak values occurring late in the evening (≈ 23:00) before a steady descent. This delayed acrophase suggests that, in young children, febrile dynamics may extend the thermogenic phase, potentially reflecting sustained inflammatory drive and/or age-related differences in circadian entrainment. This phase shift is clinically and biologically relevant. It may be influenced by developmental features of thermoregulatory control and by behavioural factors such as sleep timing and evening activity. Importantly, these findings indicate that although the fever curve is circadian in form, it is not merely a vertically shifted version of the healthy rhythm; rather, it appears phase-shifted and likely shaped by interactions between illness-related immune activation and endogenous circadian regulation.

### Age-related differences in fever pattern

**Fig. 2:**
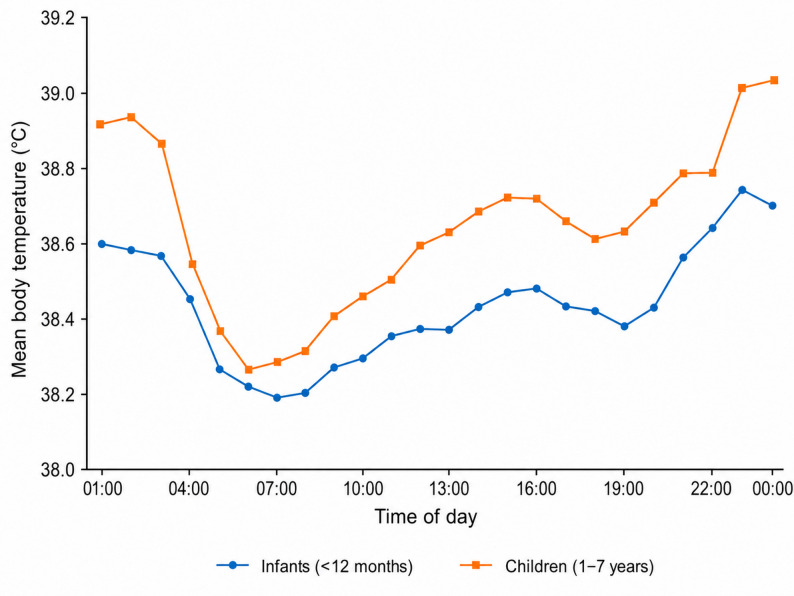
Diurnal fever curve stratified by age group. Entry-level mean hourly body temperature (local time) stratified by age: infants < 12 months (rectal measurements) versus children aged 1–7 years (tympanic measurements). Lines represent aggregate registry-level means and are not cluster-corrected child-level trajectories

We next examined diurnal fever trajectories stratified by age (Fig. 2). Figure 2 depicts entry-level mean 24-hour temperature curves for infants (< 12 months) and older children (1–7 years). In both strata, the aggregate diurnal organisation of the fever curve was maintained, with an early-morning nadir and a late-evening peak.

Across most hours, infants exhibited lower entry-level mean temperatures than older children. Differences were smallest around the morning trough (e.g., 06:00: 38.22 °C vs. 38.26 °C) and more pronounced at other times, with a maximum hourly difference of approximately 0.37 °C. Detailed hourly descriptive estimates for both age groups are provided in Table 3. During the early morning hours (approximately 04:00–06:00), the curves largely converged. These age-stratified curves are descriptive summaries of recorded entries and should not be interpreted as evidence of an individual-level developmental effect.

**Table 3:** Hourly mean temperature by age group (< 12 months vs. 1–7 years)

Time	Infants (< 12 months)			Children (1–7 years)		
	Mean (SD), °C	95% CI, °C	*N*	Mean (SD), °C	95% CI, °C	*N*
1:00 AM	38.60 (0.83)	38.52–38.68	451	38.94 (0.87)	38.88–39.00	851
2:00 AM	38.59 (0.92)	38.50–38.68	414	38.96 (0.90)	38.90–39.03	745
3:00 AM	38.57 (0.87)	38.48–38.65	396	38.87 (0.90)	38.80–38.93	747
4:00 AM	38.46 (0.88)	38.39–38.53	612	38.54 (0.97)	38.49–38.59	1,671
5:00 AM	38.27 (0.86)	38.22–38.32	1,250	38.36 (0.95)	38.33–38.39	3,703
6:00 AM	38.22 (0.83)	38.18–38.25	1,675	38.26 (0.96)	38.24–38.29	5,103
7:00 AM	38.19 (0.86)	38.15–38.23	1,756	38.28 (0.96)	38.26–38.31	5,314
8:00 AM	38.20 (0.83)	38.16–38.24	1,677	38.31 (0.95)	38.29–38.34	4,627
9:00 AM	38.27 (0.85)	38.23–38.31	1,467	38.40 (0.95)	38.37–38.43	4,152
10:00 AM	38.29 (0.85)	38.25–38.33	1,422	38.46 (0.93)	38.44–38.49	4,389
11:00 AM	38.35 (0.83)	38.30–38.39	1,437	38.50 (0.95)	38.47–38.53	4,577
12:00 PM	38.37 (0.84)	38.32–38.41	1,478	38.61 (0.93)	38.58–38.63	4,693
1:00 PM	38.37 (0.85)	38.33–38.42	1,397	38.63 (0.93)	38.61–38.66	5,108
2:00 PM	38.43 (0.82)	38.39–38.47	1,496	38.69 (0.91)	38.67–38.72	5,088
3:00 PM	38.47 (0.84)	38.43–38.51	1,501	38.73 (0.89)	38.71–38.76	4,971
4:00 PM	38.48 (0.79)	38.44–38.51	1,628	38.73 (0.90)	38.70–38.75	5,600
5:00 PM	38.43 (0.77)	38.40–38.47	2,063	38.66 (0.89)	38.64–38.68	7,118
6:00 PM	38.42 (0.79)	38.39–38.45	2,128	38.61 (0.92)	38.59–38.63	7,276
7:00 PM	38.38 (0.84)	38.34–38.42	1,723	38.63 (0.94)	38.60–38.65	5,768
8:00 PM	38.42 (0.89)	38.37–38.46	1,428	38.71 (0.96)	38.68–38.74	4,316
9:00 PM	38.56 (0.94)	38.50–38.62	962	38.78 (0.99)	38.74–38.81	3,042
10:00 PM	38.64 (0.89)	38.58–38.71	624	38.78 (1.02)	38.73–38.82	1,807
11:00 PM	38.75 (0.84)	38.68–38.82	550	39.01 (0.92)	38.96–39.06	1,182
12:00 AM	38.70 (0.84)	38.62–38.77	469	39.03 (0.91)	38.97–39.08	1,023

Values are mean (SD) and 95% confidence intervals (CI) in °C. Infants (< 12 months) were measured rectally; children aged 1–7 years were measured tympanically. These measurement routes are collinear with age group and should be considered when interpreting between-group differences

*Abbreviations*: *CI* confidence interval, *N* number of temperature entries per hourly bin, *SD* standard deviation

These differences are likely multifactorial. Older children may mount more pronounced febrile responses in the context of evolving innate and adaptive immune function during early life [15–17]. In contrast, flatter profiles in infants may reflect distinct thermoregulatory characteristics in early infancy together with greater caregiver involvement in thermal support and earlier supportive measures, including antipyretic use [14, 18, 19]. Caregiver beliefs and thresholds for measuring and treating fever may further shape documentation behaviour, particularly in very young children [2, 20].

Despite differences in magnitude, the timing of the diurnal rhythm was consistent across age strata. In both groups, mean temperatures were lowest in the early morning (around 06:00–07:00) and highest late in the evening (around 23:00). Importantly, a lower febrile response in infants should not be interpreted as lower illness severity. Clinically, in very young infants, serious bacterial infection can occur even with modest temperature elevations [21].

### Effect of antipyretic medication on fever profile

**Fig. 3:**
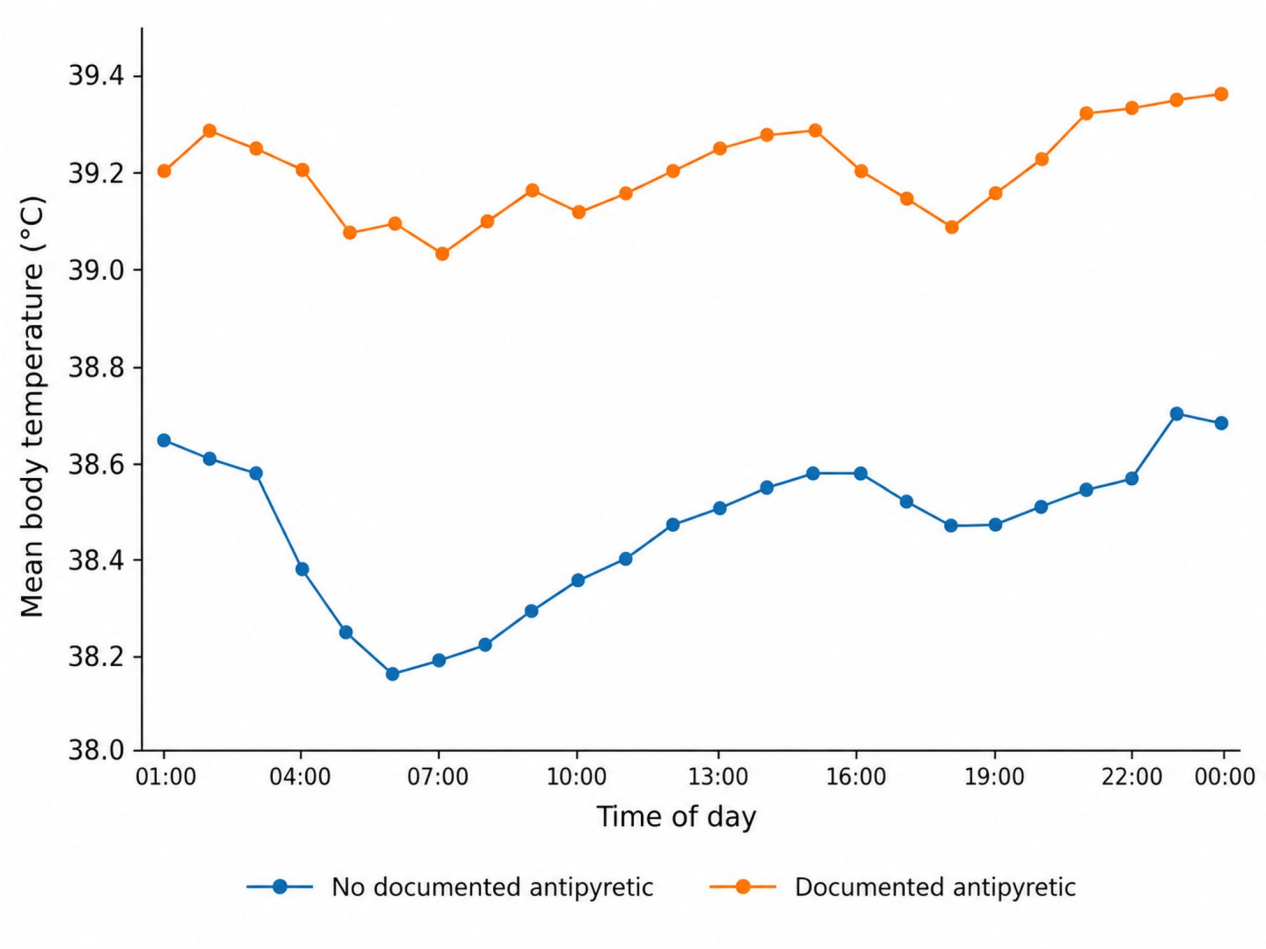
Diurnal fever curve stratified by documented antipyretic administration. Entry-level mean hourly body temperature (local time) for recordings with a documented antipyretic entry within ± 1 h of the temperature measurement versus recordings without documented antipyretic use. Lines represent aggregate registry-level means

We next analysed temperature recordings stratified by documented antipyretic administration. Figure 3 displays entry-level mean 24-hour temperature curves for measurements with a recorded antipyretic entry within ± 1 h of the temperature record, most commonly paracetamol or ibuprofen, compared with measurements without a recorded antipyretic. The comparison is descriptive and should not be interpreted as estimating the pharmacological effect of antipyretics.

Across the 24-hour cycle, mean temperatures remained consistently higher in the antipyretic stratum (Fig. 3). For example, at 21:00 the mean temperature was 39.33 °C among entries with documented antipyretic use compared with 38.55 °C among entries without recorded antipyretics, corresponding to an observed mean difference of 0.78 °C. Differences of similar magnitude (approximately 0.6–1.0 °C) were observed across most hours (Table 4).

**Table 4:** Hourly mean temperature by antipyretic use

Time	No antipyretics			With antipyretics			Comparison		
	Mean (SD), °C	95% CI, °C	*N*	Mean (SD), °C	95% CI, °C	*N*	Mean diff., °C	SE	95% CI, °C
1:00 AM	38.64 (0.82)	38.57–38.71	566	39.20 (0.74)	39.13–39.27	405	0.56	0.05	0.46–0.66
2:00 AM	38.59 (0.90)	38.51–38.67	484	39.29 (0.69)	39.22–39.36	387	0.70	0.06	0.59–0.81
3:00 AM	38.56 (0.88)	38.48–38.63	531	39.24 (0.76)	39.16–39.32	323	0.68	0.06	0.57–0.79
4:00 AM	38.35 (0.89)	38.29–38.40	1,130	39.21 (0.75)	39.13–39.28	400	0.86	0.05	0.76–0.96
5:00 AM	38.21 (0.86)	38.18–38.24	2,611	39.06 (0.78)	39.00–39.11	668	0.85	0.04	0.78–0.92
6:00 AM	38.13 (0.85)	38.10–38.15	3,711	39.09 (0.78)	39.03–39.15	729	0.96	0.03	0.90–1.02
7:00 AM	38.15 (0.87)	38.12–38.18	3,778	39.02 (0.80)	38.96–39.07	756	0.87	0.03	0.81–0.93
8:00 AM	38.18 (0.86)	38.15–38.21	3,482	39.09 (0.75)	39.03–39.15	593	0.91	0.03	0.85–0.97
9:00 AM	38.25 (0.88)	38.22–38.28	3,077	39.16 (0.78)	39.10–39.22	631	0.91	0.04	0.84–0.98
10:00 AM	38.33 (0.85)	38.30–38.36	3,127	39.11 (0.75)	39.06–39.17	739	0.78	0.04	0.71–0.85
11:00 AM	38.36 (0.87)	38.33–38.39	3,236	39.16 (0.77)	39.10–39.21	854	0.80	0.04	0.73–0.87
12:00 PM	38.45 (0.86)	38.42–38.48	3,284	39.21 (0.78)	39.16–39.26	914	0.76	0.03	0.70–0.82
1:00 PM	38.48 (0.87)	38.45–38.51	3,477	39.26 (0.75)	39.21–39.30	905	0.78	0.02	0.74–0.82
2:00 PM	38.54 (0.84)	38.51–38.57	3,461	39.28 (0.76)	39.23–39.33	959	0.74	0.02	0.70–0.78
3:00 PM	38.58 (0.84)	38.55–38.60	3,394	39.29 (0.76)	39.24–39.33	985	0.71	0.02	0.67–0.75
4:00 PM	38.58 (0.82)	38.56–38.61	3,691	39.21 (0.74)	39.17–39.25	1,295	0.63	0.02	0.59–0.67
5:00 PM	38.51 (0.81)	38.48–38.53	4,700	39.14 (0.73)	39.11–39.18	1,843	0.63	0.02	0.59–0.67
6:00 PM	38.45 (0.86)	38.42–38.47	4,768	39.09 (0.76)	39.05–39.12	1,858	0.64	0.02	0.60–0.68
7:00 PM	38.45 (0.88)	38.43–38.48	3,943	39.16 (0.77)	39.12–39.20	1,288	0.71	0.02	0.67–0.75
8:00 PM	38.50 (0.89)	38.46–38.53	2,932	39.23 (0.82)	39.18–39.28	997	0.73	0.04	0.66–0.80
9:00 PM	38.55 (0.93)	38.51–38.59	1,787	39.33 (0.79)	39.28–39.38	902	0.78	0.04	0.71–0.85
10:00 PM	38.55 (0.95)	38.50–38.61	1,102	39.31 (0.79)	39.25–39.37	665	0.76	0.04	0.68–0.84
11:00 PM	38.72 (0.88)	38.65–38.78	737	39.33 (0.71)	39.27–39.39	567	0.61	0.04	0.53–0.69
12:00 AM	38.69 (0.90)	38.62–38.76	582	39.34 (0.74)	39.27–39.40	506	0.65	0.05	0.55–0.75

Values are mean (SD) and 95% confidence intervals (CI) in °C. Mean differences are shown as ‘with antipyretics’ minus ‘no antipyretics’. Standard errors (SE) and 95% CIs for the mean difference were derived from within-hour SEs of the two strata using a normal approximation. The antipyretic stratum captures entries within ± 1 h of a documented antipyretic administration; the comparison is observational and should not be interpreted as a causal estimate of antipyretic effect

*Abbreviations*: *CI* confidence interval, *N* number of temperature entries per hourly bin, *SD* standard deviation, *SE* standard error

In addition, the operational definition of antipyretic exposure (a medication entry within ± 1 h of the temperature record) captures both pre- and post-dosing windows. Consequently, elevated temperatures measured shortly before administration, or after waning of effect, may be classified within the “antipyretic” stratum. Accordingly, this comparison does not estimate a causal antipyretic effect but reflects an observational grouping within a naturalistic dataset. Importantly, the circadian structure of fever was preserved in both strata. Despite clear differences in overall temperature level, the no-antipyretic stratum showed a diurnal pattern consistent with the overall sample; the antipyretic stratum showed a comparatively attenuated pattern.

Finally, user interaction patterns in FeverApp suggest that antipyretics are most administered in the late afternoon or early evening, coinciding with periods of higher fever intensity and perceived discomfort, whereas overnight dosing appears less frequent, likely reflecting avoidance of sleep disruption unless temperatures are considered concerning. Current guidelines indeed recommend not to awake a child to measure fever or to give an antipyretic [22]. This behavioural timing may further contribute to the pronounced afternoon/evening separation between medicated and non-medicated recordings.

### Frequency of high fever by time of day

To further evaluate the clinical relevance of the diurnal fever rhythm, we analysed the time-of-day distribution of high-grade fever descriptively. Among 121,364 temperature recordings included in the threshold analysis, 41,859 (34.5%) were ≥ 39.0 °C (high fever) and 7,591 (6.3%) were ≥ 40.0 °C (very high fever). The observed proportion of high-fever measurements varied substantially across the day and was lowest in the early morning (05:00–08:59, 24.8% ≥39 °C; 3.6% ≥40 °C). The highest proportions were observed during the late evening/night (21:00–00:59, 46.6% ≥39 °C; 10.6% ≥40 °C).

The observed diurnal pattern has direct clinical implications. A child who presents with only mild to moderate fever in the morning may still develop higher temperatures later the same day; therefore, a low morning value should not be interpreted as definitive reassurance in isolation. Conversely, high fever during the early morning hours appeared comparatively uncommon in the aggregated recordings and may justify careful clinical assessment when accompanied by concerning symptoms or impaired general condition. Time of day should not be used as a stand-alone diagnostic marker, but it can complement assessment of age, general condition, warning signs, and clinical course.

## Discussion

In this app-based observational study comprising more than 122,000 temperature recordings in young children, we describe a clear aggregate time-of-day pattern in febrile temperature entries. To our knowledge, this represents one of the largest real-world datasets describing diurnal fever patterns in children outside clinical settings. By providing high-resolution, home-based measurements, the study adds descriptive granularity at the interface of chronobiology, pediatrics, and digital health.

We identified a consistent aggregate diurnal fever rhythm characterised by lower recorded temperatures in the early morning and a progressive rise toward a late-evening peak. With an observed trough-to-peak difference of approximately 0.68 °C, the entry-level curve indicates that a comparatively modest fever reading in the early morning may be compatible with higher temperatures later in the day, without necessarily reflecting disease progression alone [5, 6, 23]. This time dependency challenges overly static fever interpretation: fixed thresholds (e.g., ≥ 38.0 °C), while operationally useful, do not account for circadian variation and may contribute to underrecognition in the morning and overinterpretation later in the day [24]. Fever assessment should therefore be contextualised, integrating time of day and trajectory rather than relying on isolated measurements [5, 23].

It is also worth noting that focal bacterial infections may blunt or override typical circadian modulation of fever, potentially contributing to early-morning high-fever recordings. This hypothesis is consistent with existing evidence: fever height alone is a poor predictor of serious bacterial infection, particularly in infants and toddlers, and the German S3 guideline on fever management explicitly cautions against interpreting fever severity primarily through temperature thresholds, emphasising the child’s general condition and warning signs instead [22]. Whether atypical circadian fever profiles (e.g., persistent high-grade fever in the early morning) carry additional diagnostic signal warrants validation in cohorts with microbiological linkage. Because physician-confirmed diagnoses were not available, we cannot determine whether diurnal patterns differed between uncomplicated upper respiratory tract infections and severe bacterial infection or sepsis; this interpretation remains hypothesis-generating.

The entry-level mean curves suggested age-related differences in recorded fever intensity, while the overall timing of the diurnal pattern was similar across age strata. This pattern is congruent with pediatric experience and biologically plausible mechanisms, including developmental differences in immune signaling, thermoregulation, and caregiver response behaviour (e.g., earlier supportive measures or antipyretic use) [16, 17]. However, because age group was structurally linked to measurement site and because the data were analysed as heterogeneous entry-level aggregates, these differences should be interpreted cautiously. For example, a temperature of 38.2 °C in a 2-month-old may have different diagnostic implications than 39.5 °C in an otherwise well preschool child with a minor viral illness [25]. These nuances are relevant for caregiver counselling and are expressed in the new German guideline on fever management in children and youth [22].

Recordings being temporally associated with antipyretic administration were linked to higher mean temperature profiles throughout the day. This is most consistent with confounding by indication: caregivers tend to administer medication in response to higher fever and/or greater discomfort, rather than medication being ineffective. Antipyretic use did not abolish the circadian structure. Early-morning lows and evening peaks persisted in medicated children. This observation suggests that circadian regulation of thermoregulation and immune function remains largely intact during febrile illness and is only transiently modulated by pharmacological intervention. It also raises a clinically relevant chronotherapeutic question: should timing of antipyretic use be considered more deliberately? If a child is comfortable during the morning circadian low, watchful waiting may be reasonable, whereas later-day administration could potentially mitigate the expected evening rise. However, newer guidelines agree that antipyretics should not be given with the sole aim of reducing fever but are rather to be seen as pain killers to alleviate significant pain or discomfort [4].

The observed time-of-day dependence of high fever has implications for triage and caregiver behaviour. In the entry-level threshold analysis, ≥ 39 °C and ≥ 40 °C recordings were least frequent in the early morning and most frequent in the late evening/night. Recognising the expected late-day rise may reduce unnecessary alarm and inappropriate care-seeking, while high fever at an atypical circadian phase should be interpreted together with warning signs, general condition, age, and clinical course. Triage algorithms and app-based guidance could benefit from explicit circadian framing, but time-of-day-adapted thresholds require prospective validation before clinical implementation.

Finally, this study demonstrates the feasibility and utility of participatory, app-based research in pediatrics. Through FeverApp, we captured frequent, context-rich, time-stamped data from thousands of families in real-world home settings. This ecological momentary assessment (EMA) approach enables the analysis of temporal symptom dynamics, reduces recall bias, and supports detailed chronobiological evaluation of febrile illness [10, 11]. Beyond methodological advantages, the platform may strengthen parental engagement and health literacy by combining evidence-based guidance with structured data contribution. The reliability of the registry has been supported in prior work, including concordance between caregiver-reported and clinically validated fever episodes and consistent medication documentation [10]. These observations align with broader EMA evidence in children demonstrating good adherence and parent-child agreement in mobile symptom tracking [26], and with participatory digital health models indicating feasibility and ethical robustness across youth health domains [27]. In practical terms, this means that FeverApp-derived temperature values are embedded in a broader caregiver-reported context, including symptoms, medication use, and healthcare contacts, even though the present analysis focused primarily on temperature timing and antipyretic exposure.

Future developments should examine whether app-based fever registries can be linked with validated non-invasive continuous temperature monitoring devices. Such integration could reduce caregiver-driven sampling bias, capture nocturnal and early-morning temperature dynamics more completely, and help distinguish biological fever rhythms from measurement behaviour. Evidence from pediatric oncology, haemato-oncology, oncology nursing, and intensive-care settings suggests that continuous or high-frequency temperature monitoring may support earlier fever detection in selected high-risk populations [28–33]. However, these findings should not be transferred directly to otherwise healthy children with common febrile illnesses without prospective validation.

In the longer term, FeverApp-like platforms combined with validated wearable-generated temperature series could support age-stratified, time-of-day-specific temperature maps, consistent with previous evidence supporting age-specific fever thresholds [34]. Such maps might improve parental counselling, safety-netting, and contextual interpretation of fever trajectories. Any AI-supported triage, warning system, or dose-support function would require diagnostic linkage, prospective validation, assessment of false alerts, equity safeguards, and regulatory evaluation before clinical implementation.

### Limitations

Several limitations should be considered. First, this was an app-based observational registry and not a population-based cohort. Participation, recording frequency, and timing of measurements were caregiver-driven. Families using FeverApp may differ from non-users in health literacy, concern about fever, digital access, and thresholds for measuring or treating fever [35]. Therefore, the results should be interpreted as patterns observed in a Germany-based app-using cohort rather than as representative estimates for all children.

Second, the dataset contains repeated measurements nested within fever episodes, children, and potentially families. The revised analyses were therefore restricted to descriptive entry-level summaries. Individual entries were not statistically independent, and children with frequent recordings may have contributed disproportionately to hourly means and threshold proportions. Because many fever episodes contained only few and non-equidistant temperature entries, individual time-series modelling was not feasible. Even density-based displays of the non-aggregated measurements remain entry-level visualisations and do not resolve clustering. The results should therefore be interpreted as heterogeneous aggregate patterns of recorded FeverApp measurements rather than as individual-level fever trajectories or child-level risk estimates.

Third, measurements were entered by caregivers under real-world conditions. Thermometer type, device calibration, measurement technique, and contextual factors could not be standardised. To improve comparability, analyses were restricted to rectal measurements in infants and tympanic measurements in older children; however, this introduced complete confounding between age group and measurement site. Consequently, age-related temperature differences should be interpreted cautiously.

Fourth, diagnoses, laboratory findings, microbiological results, neurological comorbidities, sleep-wake patterns, exact antipyretic dose and timing, and non-pharmacological cooling measures such as wraps or tepid sponging were not systematically available. Viral, bacterial, and undifferentiated febrile illnesses were therefore analysed together. Antipyretic comparisons are particularly vulnerable to confounding by indication, because medication is more likely to be given when fever or discomfort is already higher. These limitations preclude causal conclusions and illness-specific risk stratification.

Finally, generalisability may be limited by the geographic, cultural, and climatic context. Most users were based in Germany, a high-income setting with a temperate climate and specific healthcare-seeking norms. Future studies should link app-based temperature trajectories with clinical diagnoses, comorbidity data, and, where appropriate, validated continuous temperature monitoring.

## Conclusions

Fever in young children should be interpreted as a dynamic 24-hour pattern rather than as a single static temperature value. In this app-based observational cohort, recorded febrile temperatures followed a clear aggregate time-of-day pattern, with lower values in the early morning and higher values later in the day. This temporal context may help clinicians and caregivers interpret fever more realistically: a low morning value should not be used as reassurance in isolation, while late-day increases may partly reflect expected circadian dynamics.

The practical call to action is to integrate time-of-day awareness into fever counselling, safety-netting, and digital decision support. Future FeverApp developments should move from isolated temperature entries toward validated fever trajectories that combine age, symptoms, medication timing, diagnostic information, and, potentially, continuous wearable temperature data. Before time-adapted thresholds, AI-supported triage, or dose-support tools are implemented clinically, they require prospective validation in representative pediatric populations.

## Supplementary Information

Additional file 1: Supplementary Figure S1. Entry-level distribution of FeverApp temperature recordings by time of day. Binned density heatmap showing relative frequencies of body temperature recordings in 0.1 °C bins across each hour of the day. The dotted horizontal line marks 39.0 °C, the predefined threshold for high fever. Each temperature recording contributes equally; no weighting or clustering by child, fever episode, or family was applied.

Additional file 2.

Additional file 3.

## Data Availability

Due to data protection requirements and privacy regulations, the full dataset generated and analysed during the present study is not publicly available. A de-identified summary dataset may be made available from the corresponding author upon reasonable request and subject to appropriate institutional and ethical approvals.

## Abbreviations

EMA: Ecological momentary assessment
IQR: Interquartile range (25th–75th percentile)
