# Supplementary material for: Diurnal variations of fever in children: results from an app-based observational study
Source: BMC Pediatr. 2026 Aug 5;26:719. doi: 10.1186/s12887-026-07432-y (PMC13450515; doi:10.1186/s12887-026-07432-y)
Supplement: Supplementary file 1 [file 12887_2026_7432_MOESM1_ESM.docx]

**Supplementary Figure S1. Entry-level distribution of FeverApp temperature recordings by time of day**. Entry-level distribution of FeverApp temperature recordings by time of day. Binned density heatmap showing relative frequencies (%) of body temperature recordings in 0.1 °C bins across each hour of the day. The dotted horizontal line marks 39.0 °C, the predefined threshold for high fever. Each temperature recording contributes equally; no weighting or clustering by child, fever episode, or family was applied. The figure is descriptive and does not represent individual fever trajectories or child-level risk estimates.
