# Supplementary material for: Diurnal variations of fever in children: results from an app-based observational study
Source: BMC Pediatr. 2026 Aug 5;26:719. doi: 10.1186/s12887-026-07432-y (PMC13450515; doi:10.1186/s12887-026-07432-y)
Supplement: Supplementary file 2 [file 12887_2026_7432_MOESM2_ESM.docx]

**Supplementary Figure S1**

**Participant Flow Diagram**

Diurnal Variations of Fever in Children: Results from an App-Based Observational Study

**Figure legend:** Flow diagram of participants included in the analysis of the FeverApp registry (DRKS00016591), September 2019 – September 2023. Eligibility criteria required at least one temperature measurement ≥38.0 °C and use of the protocol-specified measurement site (rectal in infants aged <12 months; tympanic in children aged 1–7 years). Measurements obtained at other sites (axillary, oral) were excluded; exact exclusion counts were not recorded. Primary analysis comprised all 122,890 eligible measurements; age-stratified and antipyretic sub-analyses are shown as secondary analyses. For the antipyretic stratification, medication data were available for 27,108 of 28,620 episodes (1,512 missing). The high-grade fever threshold analysis (≥39.0 °C) included 121,364 measurements.

**Abbreviations:** IQR, interquartile range; DRKS, Deutsches Register Klinischer Studien (German Clinical Trials Register).

**Reporting guideline:** Prepared in accordance with the STROBE reporting guidelines for observational studies (von Elm E et al. BMJ 2007;335:806–8).

**Funding:** Software AG Foundation (20%); German Federal Ministry of Education and Research (BMBF grant 01GY1905; 80%). The funders had no role in study design, data collection, analysis, interpretation, or the decision to publish.
