# Supplementary material for: Diurnal variations of fever in children: results from an app-based observational study
Source: BMC Pediatr. 2026 Aug 5;26:719. doi: 10.1186/s12887-026-07432-y (PMC13450515; doi:10.1186/s12887-026-07432-y)
Supplement: Supplementary file 3 [file 12887_2026_7432_MOESM3_ESM.docx]

**STROBE Checklist — Cohort Study (Final Version)**

Diurnal Variations of Fever in Children: Results from an App-Based Observational Study

FeverApp Registry · DRKS00016591 (registered 7 February 2019)

Reference: von Elm E, Altman DG, Egger M, Pocock SJ, Gøtzsche PC, Vandenbroucke JP; STROBE Initiative. The Strengthening the Reporting of Observational Studies in Epidemiology (STROBE) Statement: guidelines for reporting observational studies. BMJ. 2007;335:806–8.

| **Item** | **Checklist item (cohort study)** | **Addressed in manuscript — location / comment** | **Page** |
| --- | --- | --- | --- |
| **Title and Abstract** | | | |
| 1a | Title | **Study design indicated in the title.** → The term 'observational study' appears in the manuscript title. ✓ | p. 1 |
| 1b | Abstract | **Informative and balanced summary provided.** → Structured abstract with Background, Methods (including measurement sites, study period, sample size), Results (key findings with values), Conclusions, and Trial registration. Registration number (DRKS00016591), date (7 February 2019), and URL (https://drks.de/search/en/trial/DRKS00016591) included. ✓ | p. 1 |
| **Introduction** | | | |
| 2 | Background / rationale | **Scientific background and rationale explained.** → Chronobiology of body temperature, fever management, 'fever phobia', and the evidence gap on diurnal fever patterns in children outside clinical settings described. ✓ | pp. 3–4 |
| 3 | Objectives | **Specific objectives and pre-specified hypotheses stated.** → Three hypotheses explicitly formulated: (1) circadian temperature pattern preserved during febrile illness; (2) infants exhibit lower peak temperatures than older children; (3) antipyretic use does not abolish circadian structure but follows it. ✓ | p. 5 |
| **Methods** | | | |
| 4 | Study design | **Key elements of study design presented early.** → 'Retrospective observational analysis' of an app-based registry (FeverApp, DRKS00016591) stated at the beginning of the Methods section. ✓ | p. 5 |
| 5 | Setting | **Setting, locations, and relevant dates described.** → Germany; September 2019 – September 2023 (registry launch and analysis period). DRKS registration date (7 February 2019) stated in the Abstract and the Methods. ✓ | pp. 5–6 |
| 6* | Participants (cohort) | **Eligibility criteria, sources, and methods of participant selection described.** → Children aged 0–7 years; ≥1 temperature measurement ≥38.0 °C; measurement site restricted to rectal (<12 months) or tympanic (1–7 years); period September 2019–September 2023. **Follow-up:** Statement added to the Participants section (Methods): 'As this study is based on a registry with passive ecological momentary assessment, active follow-up of individual participants was not conducted and is not applicable to this study design.' ✓* **Flow diagram (STROBE Item 13):** Created as Supplementary Fig. S1 and included as a separate submission file. ✓* | pp. 6–7 + Suppl. |
| 7 | Variables | **All outcomes, exposures, predictors, and potential confounders clearly defined.** → Primary variable: time of day of temperature recording. Secondary variables: age group (<12 months vs 1–7 years) and antipyretic use (medication entry within ±1 h of temperature measurement). ✓ | p. 7 |
| 8 | Data sources / measurement | **Sources and details of measurement described for each variable.** → App-based real-time parental entries; time-stamped to local time; temperature recorded to one decimal place; medication data from a separate linked dataset. ✓ | p. 7 |
| 9 | Bias | **Efforts to address potential sources of bias described.** → Dedicated section 'Bias and error considerations': selection bias (app users), measurement site confounding (collinear with age group), temporal sampling bias (fewer night-time recordings), and antipyretic misclassification — each described with direction and magnitude. ✓ | pp. 10-11 |
| 10 | Study size | **Explanation of how study size was arrived at.** → Sentence added to the Outcome measures and statistical analysis section (Methods): 'No a-priori sample size calculation was performed; all eligible registry entries within the study period were included in the analysis.' ✓* | p. 10 |
| 11 | Quantitative variables | **Handling of quantitative variables in the analyses explained.** → Temperature treated as a continuous variable; aggregated into hourly bins (00:00–00:59, etc.); hourly means with 95% CIs reported; odds ratios from logistic regression for binary high-fever thresholds. ✓ | pp. 7–9 |
| 12* | Statistical methods (cohort) | **Statistical methods fully described.** (a) Hourly means and CIs; logistic regression with time interval and age group as predictors. ✓ (b) Missing data: no imputation; missing values excluded and quantified (n=1,512 episodes without medication data). ✓ (c) Sensitivity analyses: alternative aggregation strategies discussed in the Limitations section. ✓ Software: IBM SPSS Statistics, version 28 (IBM Corp., Armonk, NY, USA). ✓ | pp. 7–9 |
| **Results** | | | |
| 13* | Participants | **Numbers of individuals at each stage of the study reported.** → 12,454 children; 28,620 fever episodes; 122,890 temperature measurements. Exclusion criterion (non-protocol measurement sites: axillary, oral) stated; exact exclusion count not available from registry structure. **Flow diagram created as Supplementary Fig. S1** (British English, STROBE Item 13); included as a separate submission file. ✓* | p. 10 -11+ Suppl. |
| 14* | Descriptive data (cohort) | **Characteristics of study participants described.** (a) Table 1: demographic and clinical baseline characteristics (age, sex, number of episodes and measurements, medication data). ✓ (b) Missing data: n=1,512 episodes with missing medication information explicitly stated. ✓ | pp. 11-12 |
| 15* | Outcome data (cohort) | **Outcome events or summary measures over time reported.** → Tables 2–4: hourly mean temperatures ±95% CI for the overall sample, by age group, and by antipyretic use. Time-of-day distribution of high-grade fever (≥39 °C) and very high fever (≥40 °C). Odds ratios with 95% CI from logistic regression. ✓ | pp. 12–18 |
| 16 | Main results | **Unadjusted and adjusted estimates reported with precision.** → Diurnal curve (nadir 06:00 h, 38.25 °C; acrophase 23:00 h, 38.93 °C; amplitude 0.68 °C); age-group differences; antipyretic stratification; adjusted ORs for high fever with 95% CIs. ✓ | pp. 12–18 |
| 17 | Other analyses | **Other analyses reported, including subgroup and sensitivity analyses.** → Subgroup analyses by age group and antipyretic use; exploratory seasonal analyses mentioned (results not shown). ✓ | pp. 17-18 |
| **Discussion** | | | |
| 18 | Key results | **Key results summarised with reference to study objectives.** → Discussion opens with four numbered main findings, each directly related to the pre-specified hypotheses. ✓ | pp.24-27 |
| 19 | Limitations | **Limitations discussed, including direction and magnitude of potential bias.** → Dedicated Limitations section covering: selection bias (app users); measurement site confounding (collinear with age group; direction of effect described); temporal sampling bias (fewer night recordings; likely overestimation of morning nadir); causal inference limitations; generalisability (Germany, temperate climate, sociodemographic profile of users). ✓ | pp. 27–30 |
| 20 | Interpretation | **Cautious overall interpretation of results.** → Clinical implications for fever measurement, parental counselling, and app-based decision support discussed; comparison with published literature; chronotherapeutic question framed explicitly as a hypothesis for future research. ✓ | pp. 23–27 |
| 21 | Generalisability | **Generalisability (external validity) discussed.** → Geographic restriction (Germany, temperate climate); seasonal effects explored but not marked; sociodemographic profile of app users (predominantly mothers with higher educational attainment; underrepresentation of families with lower educational attainment or migration background). ✓ | pp. 22–23 |
| **Other Information** | | | |
| 22 | Funding | **Source of funding and role of funders stated.** → Software AG Foundation (20%); German Federal Ministry of Education and Research (BMBF grant 01GY1905; 80%), 2019–2024; Witten/Herdecke University and Software AG Foundation until 2034. **Role of funders declared:** 'The funders had no role in study design, data collection, analysis, interpretation, or the decision to publish.' ✓* | Decl. |
